# Modeling vitamin B_1_ transfer to consumers in the aquatic food web

**DOI:** 10.1038/s41598-019-46422-2

**Published:** 2019-07-11

**Authors:** M. J. Ejsmond, N. Blackburn, E. Fridolfsson, P. Haecky, A. Andersson, M. Casini, A. Belgrano, S. Hylander

**Affiliations:** 1 Institute of Environmental Sciences, Jagiellonian University, ul. Gronostajowa 7, 30-387 Kraków, Poland; 2 Centre for Ecology and Evolution in Microbial Model Systems – EEMiS, Linnaeus University, 39182 Kalmar, Sweden; 3 BIORAS, Hejreskovvej 18B, Copenhagen, Denmark; 4 Department of Ecology and Environmental Science, Umeå University, SE-901 87 Umeå, Sweden; 5 Umeå Marine Sciences Centre, SE-905 71 Hörnefors, Sweden; 6 Department of Aquatic Resources, Institute of Marine Research, Swedish University of Agricultural Sciences, Turistgatan 5, 45330 Lysekil, Sweden; 7 Swedish Institute for the Marine Environment (SIME), University of Gothenburg, Box 260, SE-405 30 Gothenburg, Sweden

**Keywords:** Biooceanography, Ecological modelling, Ecosystem ecology, Marine biology, Environmental impact

## Abstract

Vitamin B_1_ is an essential exogenous micronutrient for animals. Mass death and reproductive failure in top aquatic consumers caused by vitamin B_1_ deficiency is an emerging conservation issue in Northern hemisphere aquatic ecosystems. We present for the first time a model that identifies conditions responsible for the constrained flow of vitamin B_1_ from unicellular organisms to planktivorous fishes. The flow of vitamin B_1_ through the food web is constrained under anthropogenic pressures of increased nutrient input and, driven by climatic change, increased light attenuation by dissolved substances transported to marine coastal systems. Fishing pressure on piscivorous fish, through increased abundance of planktivorous fish that overexploit mesozooplankton, may further constrain vitamin B_1_ flow from producers to consumers. We also found that key ecological contributors to the constrained flow of vitamin B_1_ are a low mesozooplankton biomass, picoalgae prevailing among primary producers and low fluctuations of population numbers of planktonic organisms.

## Introduction

Vitamin B_1_ (thiamin) is necessary for the proper functioning of the majority of organisms because it serves as a cofactor that associates with a number of enzymes involved in primary carbohydrate and amino acid metabolism^1^. Vitamin B_1_ deficiency compromises mitochondrial functioning^2^ and causes nerve signaling malfunction in animals^3^. On a systemic level, low vitamin B_1_ levels translate into impaired health, immunosuppression and reproductive failures^4^. Synthesis of vitamin B_1_ is regulated by specific biosynthetic pathways for prokaryotes, plants and fungi^5^. All animals, as well as many prokaryotes and unicellular eukaryotes, cannot synthesize vitamin B_1_ and therefore must acquire it from exogenous pools^6,7^. Over the recent decades, diverse taxonomic groups, including piscivorous fish and sea birds, have suffered episodic events of reproductive failure and elevated mortality associated with sub-optimal vitamin B_1_ contents and characteristic symptoms of paralysis^8–14^. These reported reproductive failures in some years create demographic generational gaps in the Baltic population of the Atlantic salmon (*Salmo salar*) with mass mortality of offspring in the yolk-sac fry stage^15,16^. Vitamin B_1_ deficiency has been identified as an emerging conservation issue and a potential cause of population decline in marine animals^4^. The problem of population viability related to vitamin B_1_ deficiency concerns marine and freshwater ecosystems of the Northern Hemisphere, including the North American Great Lakes, the New York Finger Lakes and the Baltic Sea^10,11,15,17^.

The drivers of vitamin B_1_ deficiency in marine organisms are currently unknown, and therefore, there is an urgent need for a general understanding of how vitamin B_1_ is transferred through aquatic food webs. Prokaryotes and photoautotrophs are the main producers of vitamin B_1_ in marine systems^18^, but many of these unicellular taxa, both prokaryotes and eukaryotes are vitamin B_1_ auxotrophs that rely on external intake of dissolved vitamin B_1_ or its precursor compounds^6,7,18–22^. Concentrations of dissolved vitamin B_1_, its precursors and its degradation products are generally low (picomolar range), but data from natural systems are relatively scarce^18,23–26^. Recent studies suggest that biotic interactions and vitamin cycling during the process of obtaining B_1_ for cellular processes are likely affecting aquatic microbial community dynamics and composition^23,27–30^. Unicellular taxa producing or taking up dissolved vitamin B_1_ (or its precursors) are diverse with respect to cell size and mass-specific vitamin B_1_ concentration^7,31–33^, but the mechanisms that contribute to the overall dynamics of B_1_ and its precursors in the microbial realm are not well known^18,23^. In this model, we therefore consider bacteria and photoautotrophs as sources of vitamin B_1_, disregarding whether they synthesize the vitamin internally or obtain it via dissolved sources and focused on the transfer of B_1_ up the food web to consumers. These unicellular taxa are consumed by a range of proto- and metazoans of different sizes, and in turn, vitamin B_1_ flows to upper level consumers such as small planktivorous fish. The mass-specific vitamin B_1_ levels in planktivorous fish vary significantly in a spatiotemporal manner^13,34,35^, but it is unclear to what extent this results from constrained vitamin B_1_ flow from lower trophic levels. A climate change-driven increase in freshwater discharge can reduce the primary production, negatively affecting the efficiency of biomass transfer (see, e.g.^36^), and potentially constrain the flow of vitamin B_1_. Increased nutrient load due to anthropogenic activity can trigger shifts in the size distribution of planktonic producers toward smaller cells^37,38^. A shift in primary producers toward picoplankton can also potentially affect the flow of vitamin B_1_ due to increased decay of the vitamin during subsequent trophic interactions. Our work identifies conditions responsible for the constrained flow of vitamin B_1_ from unicellular organisms that are a source of vitamin B_1_ to planktivorous fishes.

One of the marine systems in the Northern Hemisphere most affected by vitamin B_1_ deficiency is the Baltic Sea, one of the best-monitored ecosystems worldwide among coastal marine areas^39,40^. The Baltic Sea is under the pressure of both anthropogenic factors and climate change. Anthropogenic activities such as agriculture have affected this system causing very high nutrient deposition rates, while past cod overfishing has caused trophic cascades with increasing abundances of planktivorous fish^41^. Apart from anthropogenic pressure, climate-change-driven increased precipitation may increase river loads of particles and dissolved organic and inorganic substances^42^. The increased discharge of dissolved substances is forecasted to affect light and biotic conditions of the sea and in turn suppresses phytoplankton biomass production and shift the carbon flow toward microbial heterotrophy^36,42^. Unicellular taxa producing or taking up dissolved vitamin B_1_ (or its precursors) in the Baltic Sea are very diverse with respect to taxonomy, cell size and mass-specific vitamin B_1_ content^7,31–33^. In general, those with smaller cells have higher carbon-specific vitamin B_1_ levels than those with large cells^31^. High nutrient loads and changes in light attenuation due to elevated inputs of riverine soluble substances and anthropogenic pressures are expected to affect the species composition of primary producers^43^. In turn, this is expected to affect the size spectra of vitamin B_1_-containing unicellular taxa in the Baltic Sea. As a general trend, the phytoplankton size spectrum in the Baltic Sea has shifted toward smaller primary producers in recent years^44^, but the effect on vitamin B_1_ provisioning to zooplankton, fish and other consumers is unknown. The transfer of vitamin B_1_ from planktivorous fish to top predators and the concentration in these organisms have been extensively studied^10,13,34,35^. The occurrence of a vitamin B_1_ deficiency syndrome called M74 in salmonids has been correlated with diet quality in the case of salmonids from Great Lakes of North America^10^ and the high abundance of a small planktivorous fish, European sprat (*Sprattus sprattus*), in the case of the Baltic Sea^13,35^. The requirements for vitamin B_1_ increase with the energy density of the food source, and the vitamin requirements of cold-water species such as salmon are often higher compared to those of warm-water species^45^. The small sprat eaten by the salmon in the Baltic Sea are energy rich (high-lipid) and hence provide a diet which is low in vitamin B_1_ per an energy unit^12,35^. The occurrence of B_1_ deficiency in North American salmonids is correlated with the abundance of a planktivorous fish called the alewife (*Alosa pseudoharengus*)^10^. The mechanism suggested to induce vitamin B_1_ deficiency is a high activity of a vitamin B_1_ degrading enzyme called thiaminase I in the alewife^10,46^. Comparable thiaminase I activities have also been detected in Baltic Sea clupeids^47^. However, there are no empirical studies that have disentangled the relative effects of vitamin B_1_-degrading enzymes and the vitamin concentration in clupeids regulating the overall vitamin B_1_ transfer to Baltic salmon. There are also studies showing that the diet of Baltic salmon in fact had a lower proportion of sprat during high M74 incidence years in the 1990s as compared to a low incidence period in 1959–1962^48^. While vitamin B_1_ deficiency in salmon can be related to the consumption of sprat in the Baltic Sea, other species, such as the omnivorous herring gull (*Larus argentatus*) and common eider ducks (*Somateria mollissima*), feeding on benthic organisms have also been suffering the consequences of critically low vitamin B_1_ levels in recent years^8,9,14^. To identify the drivers of the seasonal and yearly variation in vitamin B_1_ concentration in planktivorous fish and top consumers, we need to understand the transport pathways of this essential substance from the unicellular planktonic taxa up to the different parts of the aquatic food web.

Here, we present a model of nutrients and vitamin B_1_ flow through an aquatic food web, exemplified by the Baltic Sea, and the resulting levels of vitamin B_1_ in a key level of consumers, i.e., planktivorous fish. The model manipulates nutrient input, the abundance of planktivorous fish and the degree of light attenuation caused by dissolved compounds to seek conditions leading to the constrained flow of vitamin B_1_ from unicellular organisms to fish. Our study reveals the potential link between vitamin B_1_ deficiencies, the anthropogenic pressure of increased nutrient input, differences in stock size of planktivorous fish, and an increase in light attenuation by dissolved substances transported to marine coastal systems.

## Materials and Methods

### The model

**Figure 1:**
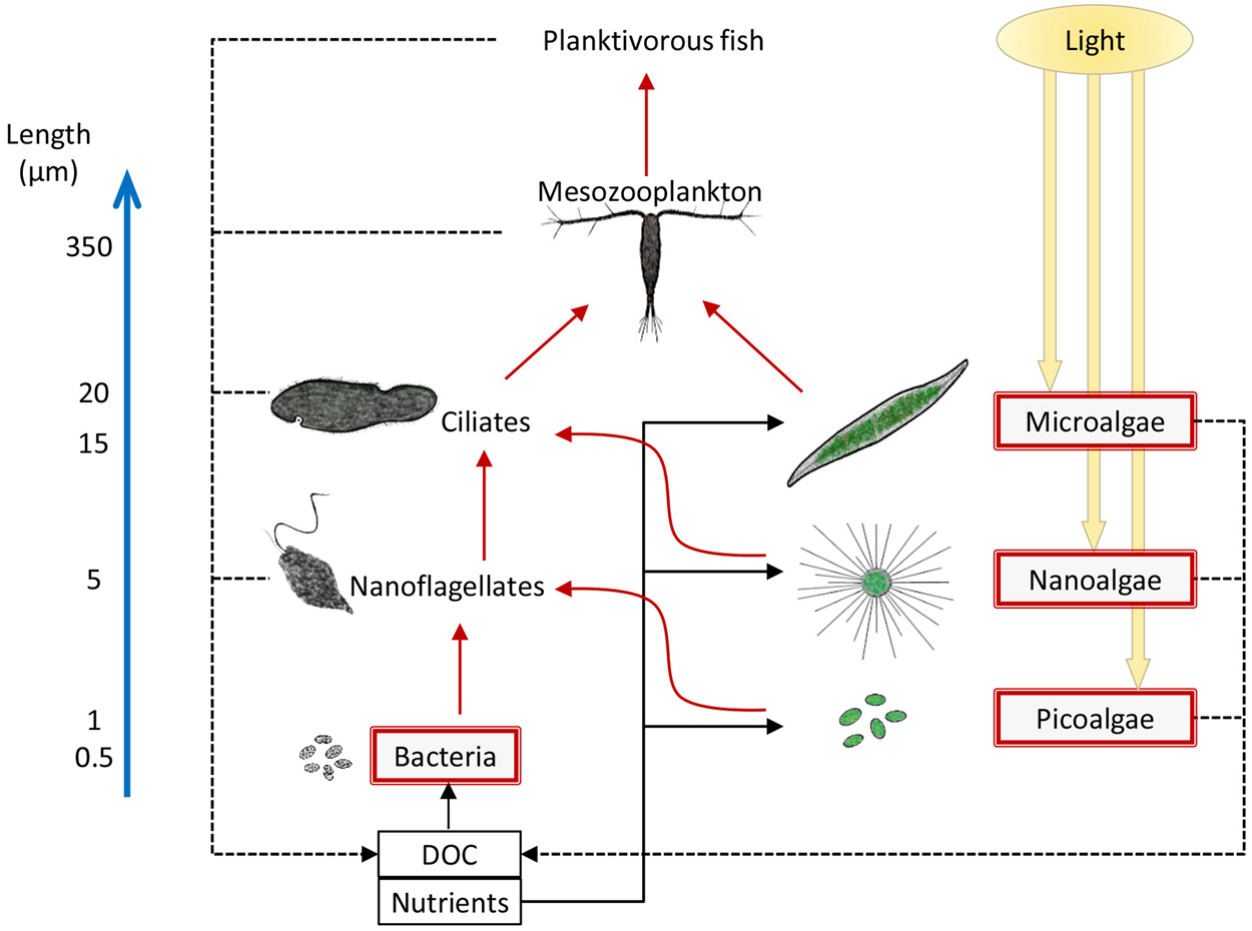
Schematic view of the modeled food web. Primary producers grow utilizing nutrients and light, whereas heterotrophic bacteria assimilate inorganic nutrients and consume dissolved organic carbon (DOC) excreted by primary producers and consumers. Organisms in each size class are grazed by consumers positioned one size class above: nanoflagellates, ciliates, mesozooplankton or planktivorous fish. The size structure of organisms in the food web is indicated along the blue arrow. Uptake, grazing or consumption (solid lines); excretion (dot-dash lines). Unicellular taxa providing vitamin B_1_ to higher trophic levels (either by uptake of dissolved vitamin B_1_, its precursors or by internal production) are marked by fields with a red outline. Black arrows indicate the flow of macronutrients. Red arrows indicate the flow of macronutrients and vitamin B_1_.

The presented model concerns a general, size-structured aquatic food web with explicit consideration of population number and nutrient and vitamin B_1_ flow between trophic levels with several elements parameterized to resemble the conditions of the Baltic Sea. The model considers pools of carbon, nitrogen, phosphorus and vitamin B_1_ to be independent and changing due to physical reactions, the physiology of organisms and population dynamics. The modeled trophic groups represent heterotrophic bacteria, primary producers (picoalgae, nanoalage and microalgae), protozoans (nanoflagellates and ciliates), mesozooplankton and small (ca. 15 cm long) planktivorous clupeid fish, with predators feeding on organisms one size level below their own (Fig. 1). The model predicts the response of the vitamin B_1_ concentration in planktivorous fish with constant population size to various scenarios of nutrient input and light attenuation caused by dissolved substances (see below). Due to the modeled half-year time frame (see below), we assume balanced birth and death rates in planktivorous fish, i.e., constant population size, which removes the need for consideration of complex multiyear trends in population age and size structure. The biomass of the other modeled groups in the food web (see Fig. 1) results from the balance between growth, respiration and death due to herbivory or predation, although we do not consider nonconsumptive mortality. Organisms live in a homogeneous, 10 m deep layer of water with instantaneous mixing and no stratification. Light intensity attenuates along with depth and fluctuates in the annual and day-night cycle (see Appendix 1 in Supporting Information) to represent conditions at the geographic location of Linnaeus Microbial Observatory (56.93°N and 17.06°E), which we also used to set nutrient input scenarios in our model (see below).

Population growth of primary producers in our model depends on available light and nutrients. The way we model light attenuation follows an approach based on Lambert-Beer’s law, adopted in theoretical studies on algae primary production (see^49^ and SI Appendix 1). The light attenuation *k* [m^−1^] in our model is determined by self-shading and the presence of dissolved organic and inorganic substances according to

1 $$k={k}_{s}{W}_{PP}+{k}_{bg}$$

where *k*_*s*_ [m^2^·mg C^−1^] sets the degree of light attenuation by algae carbon biomass *W*_*PP*_ [mg C·m^−3^] (see SI Appendix 1 for parameterization) and *k*_*bg*_ [m^−1^] is the background attenuation coefficient. We manipulated the background attenuation coefficient *k*_*bg*_ to simulate scenarios with various amounts of dissolved substances. Strong light attenuation by dissolved substances reduces the overall primary production, but it also causes large primary producers to have lower rates of light absorption per unit of volume than small phytoplankton (see^50^). Similarly, in our model, strong light attenuation by dissolved substances constrains the growth rate of large primary producers to a higher degree than it constrains the growth rate of small primary producers. The rate of nutrient uptake and the nutrient-dependent growth rate of primary producers depend allometrically on cell volume^51,52^. For details of the parameterization of light- and nutrient-dependent primary production in our model, see SI Appendix 1. We modeled the aquatic food web as an enclosed system with scenarios differing by nutrient input (see below) at the start of the vegetative season. The timing of the vegetative season, between 30 March and 1 October resembles conditions in the southern part of the Baltic Sea. Apart from fish abundance, nutrient input and degree of light attenuation due to dissolved substances (see below), each scenario started from the same initial conditions. Whereas our simulations started with picomolar concentrations of vitamin B_1_ in the tissues of modeled organisms, the results remained qualitatively unchanged when the starting concentration of vitamin B_1_ was set to the maximal allowed levels (Fig. S2 in SI Appendix 1). Each type of organism was constrained by an empirically estimated maximal mass-specific concentration of vitamin B_1_ (given in [μmol·μmol-1 C]): bacteria 1.48e-7, picoalgae 1.48e-7, nanoalgae 1.18e-7, microalgae 1.18e-7, nanoflagellates 1.32e-7, ciliates 1.27e-7, mesozooplankton 1.28e-7 and planktivorous fish 1.04e-10 (see^31,32,35^ and SI Appendix 1). We excluded from the analysis two initial months (April, May) as the ca. 60-day period was long enough for planktivorous fish feeding on mesozooplankton to potentially reach realistic levels of vitamin B_1_ and for the biomass of phytoplankton to stabilize. The model measures the proportion of days when vitamin B_1_ concentration is low, i.e., drops below 6.41^−11^ [μmol·μmol C^−1^], which is an average level of vitamin B_1_ recorded in clupeids of the Baltic Sea see^35^.

The theoretical framework we use is a modified model of an aquatic food web that explicitly considers nutrient and vitamin B_1_ flow created by N. Blackburn (BIORAS, Copenhagen)^53,54^. The model concerns interactions between organisms and their physiology based on physiochemical principals and constraints; however, processes with poorly understood proximate mechanism, are represented by empirically estimated functions (see below). The details on model parameterization not described in the main text or Supporting Information (SI Appendix 1) can be found in the work by Thelaus, *et al*.^54^. The computer program used in this study is available in SI Appendix 2.

### Determinants of vitamin B1 flow rate through the food web

Vitamin B_1_ is synthesized or absorbed from water by primary producers and heterotrophic bacteria and is transported up the food web through predation or herbivory (Fig. 1). The pool of vitamin B_1_ in organism cells is subjected to constant turnover due to degradation and input processes (see below). In primary producers and heterotrophic bacteria, the input is due to synthesis or absorption from the water, whereas consumers gain vitamin B_1_ by feeding. Manipulation of the rate at which levels of vitamin B_1_ increase in cells of bacteria and phytoplankton could for example correspond to changes in the degree of auxotrophy because high microbial cycling of vitamin B_1_ potentially reduces the overall pool of the vitamin due to losses related to excretion and absorption. In our work, we present results for the net rate of increase in vitamin B_1_ levels in cells of bacteria and algae as being equal to their metabolic rate (see SI Appendix 1). However, the conclusions from our work do not change when the rate of increase of vitamin B_1_ levels in cells of bacteria and algae is set much faster or slower than the metabolic rate (Fig. S1 in Appendix 1). This means that conclusions from our model also apply to food webs, in which a large proportion of bacteria and phytoplankton rely on the absorption of dissolved vitamin B_1_ and its precursors. Similarly, the model outcomes apply to scenarios with reduced synthesis and/or uptake of vitamin B_1_ due to other factors. In consumers, the rate of vitamin B_1_ transport is dependent on the assumed volume-specific clearance rates for predators (see SI Appendix 1), prey and predator biomass and vitamin B_1_ bioavailability i.e., the fraction of the compound that is absorbed from the food. Below, we present mainly results for vitamin B_1_ bioavailability of 15%, but we also report outcomes of a sensitivity analysis with bioavailability ranging from 5 to 20% (see SI Appendix 1 for details).

### Considered scenarios

**Figure 2:**
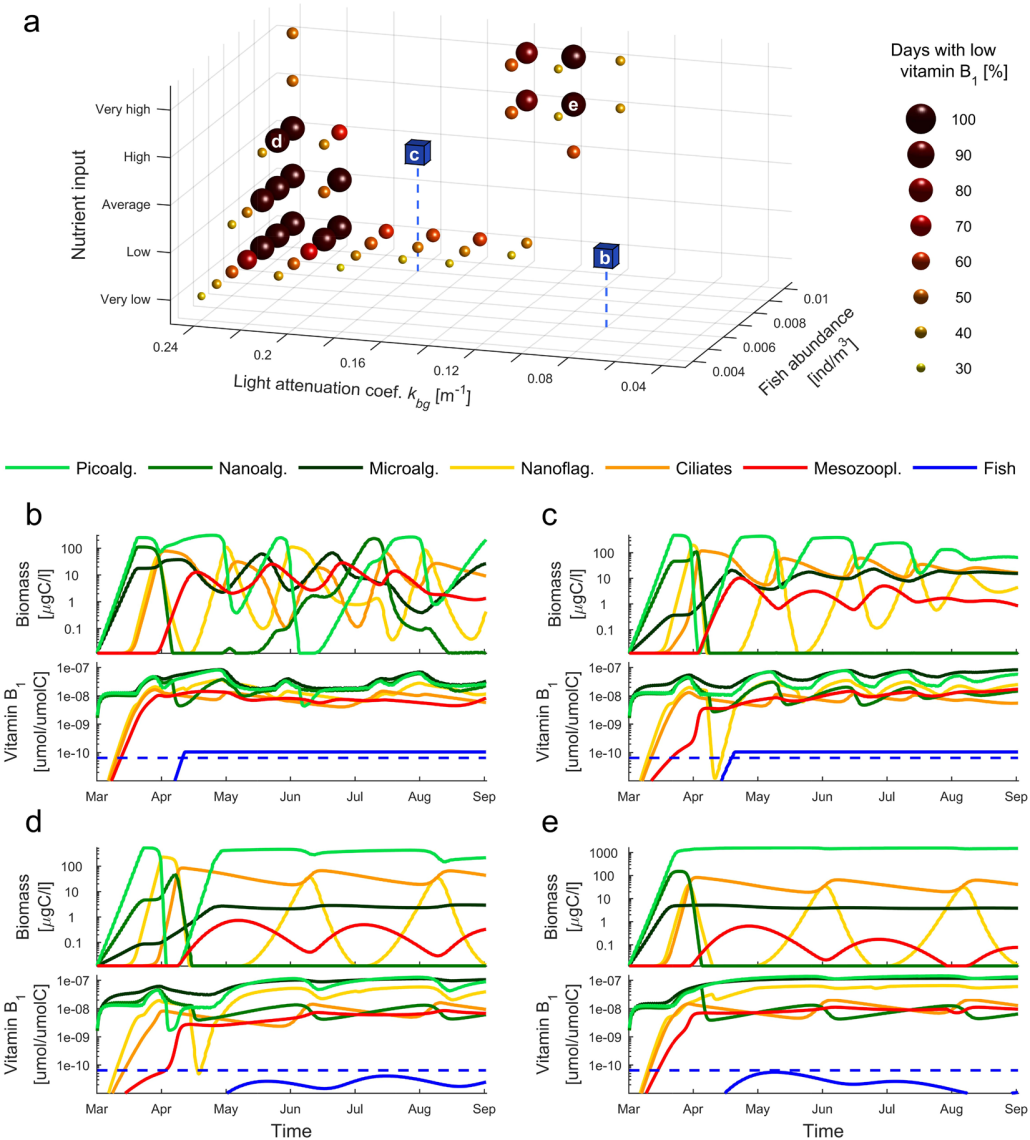
Biomass and concentrations of vitamin B_1_ in modeled organisms. (**a**) Scenarios mapped on the tested parameter space of planktivorous fish abundance, background light attenuation and nutrient input. The empty spaces match the scenarios resulting in planktivorous fish rich in vitamin B_1_, i.e., with sustained concentrations of vitamin B_1_ above the levels recorded for Baltic populations of sprat and herring for more than 70% of the analyze time period (see the main text). Blue cubes represent the position of the scenarios with fish rich in vitamin B_1_ presented also in panels b and c (letters match the scenarios presented in **b**,**c**). Spheres represent scenarios resulting in a low level of vitamin B_1_ in planktivorous fish persisting for longer than 30% of the analyzed period (see the main text). Letters indicate the position of scenarios presented in (**d**,**e**). (**b**,**c**) Example scenarios with mass-specific vitamin B_1_ levels persisting for most of the time above average levels of vitamin B_1_ recorded for Baltic populations of sprat and herring (indicated by dashed line). (**d**,**e**) Example scenarios with low mass-specific vitamin B_1_ levels persisting for most of the time during the analyzed period. (**b**–**e**) Note the difference between scenarios, (**b**–**e**) in the dynamics of fluctuations of the modeled organisms.

The model considers scenarios differing with respect to three environmental factors: (1) abundance of planktivorous fish, (2) nutrient level at the start of the vegetative season and (3) the degree to which light is attenuated by dissolved substances. (1) The fish abundance in the model directly affects the population number of mesozooplankton and, by top-down trophic cascade, also affects lower trophic levels. We used data on the abundance of Baltic populations of herring (*Clupea harengus membras*) and sprat (*Sprattus sprattus*) in 1991–2016^55^ to set a gradient of scenarios with planktivorous fish varying from low (0.004 [ind·m^−3^]) to high (0.01 [ind·m^−3^]) abundance (see SI Appendix 1 for details). (2) Our model also considers a set of scenarios varying with nutrient level at the start of the vegetative season. The assumed scenarios represent a broad range of nutrient concentrations found in the Baltic Sea, parameterized with the data extracted from the HELCOM database representing early spring nutrient concentration in the southern part of the Baltic Sea (from years 1998–2016) and data from the Linnaeus Microbial Observatory^56^. We varied the nitrogen input from a very low (2.01 μmol/l of NO_3_^−^, 0.93 μmol/l of NH_4_^+^) to a very high concentration (30 μmol/l of NO_3_^−^, 7 μmol/l of NH_4_^+^) with levels of phosphorous set relative to the nitrogen according to the Redfield ratio^57^ i.e., 16:1 (see SI Appendix 1 for details). (3) We also tested a gradient of the degree of light attenuation caused by substances dissolved in the water with a large amount of dissolved substances represented by a high background light attenuation coefficient *k*_*bg*_ (see equation 1). The constrained photon flux density in our model translates to lower rates of light absorption per unit of cell volume. Thus, a high background light attenuation coefficient *k*_*bg*_ describes a more challenging environment for large primary producers (cf. the slow growth of microalgae during the initial phase of simulations under strong light attenuation in Fig. 2c,d). We tested our model with a *k*_*bg*_ coefficient varying from 0.04 [m^−1^] to 0.24 [m^−1^] to represent a gradient of conditions ranging from very low and very high levels of substances dissolved in the water (see SI Appendix 1 for parameterization).

### Data accessibility

The computer program used in the work is available as Supplementary Information (see SI Appendix 2).

## Results

In scenarios resulting in high concentrations of vitamin B_1_ in planktivorous fish, the biomass of consumers and primary producers fluctuates over time, and over the season, different size groups of primary producers contribute to the total biomass of primary producers (Fig. 2b,c). In scenarios resulting in low vitamin B_1_ concentrations in planktivorous fish, picoalgae biomass is high and relatively stable over time; it does not drop to the minimal levels of biomass observed in scenarios with planktivorous fish rich in vitamin B_1_ for most of the simulated time (cf. picoalgae biomass in Fig. 2d,e vs. b,c). The number of days with vitamin B_1_-poor planktivorous fish is substantial in two types of scenarios: (i.) high fish abundance, high to very high nutrient input, and intermediate light attenuation, or (ii.) intermediate to high fish abundance, very low to average nutrient input, and strong background attenuation (Fig. 2a). Below, we describe the drivers and environmental correlates restricting vitamin B_1_ flow through the food web in both scenarios.

**Figure 3:**
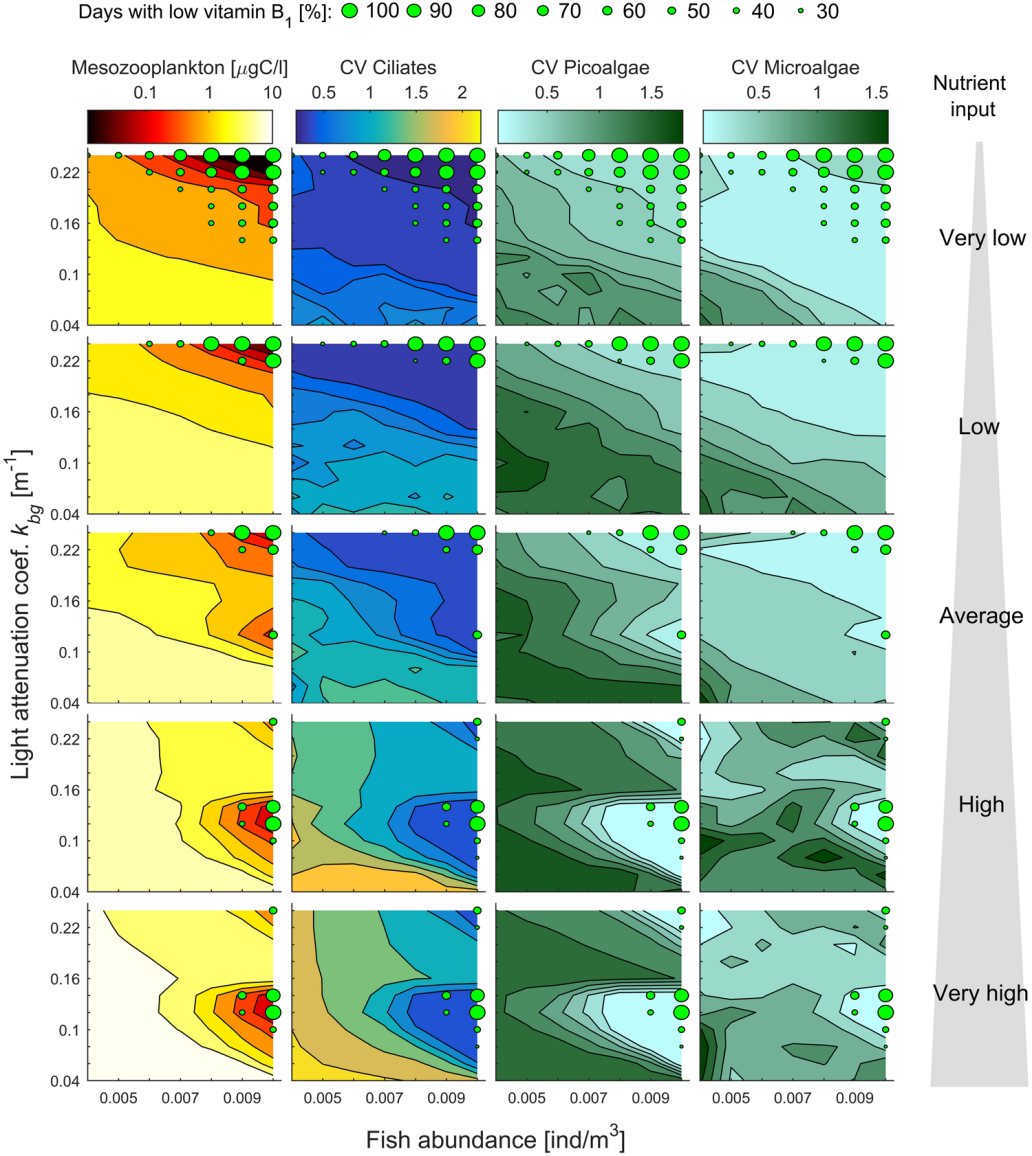
The biomass of mesozooplankton and coefficient of variation (CV) for biomass of ciliates and key groups of primary producers presented in gradients of planktivorous fish abundance, background light attenuation and nutrient input (illustrated by the gray triangular shape). The scenarios with low vitamin B_1_ levels in planktivorous fish are indicated by green circles (see the legend). (Column 1) The average biomass of mesozooplankton. (Columns 2–4) The coefficient of variation calculated for the biomass of mesozooplankton food resources (ciliates and microalgae) and picoalgae.

In both scenarios (see example scenario (i) illustrated in Fig. 2e and (ii) in Fig. 2d), the biomass of mesozooplankton is low for most of the time (compare Fig. 2d,e with b,c that represents conditions resulting in planktivorous fish rich in vitamin B_1_). Vitamin B_1_ flowing from mesozooplankton to planktivorous fish is diluted among the individuals when planktivorous fish are numerous. However, the low biomass of mesozooplankton in scenarios resulting in low vitamin B_1_ in planktivorous fish can be only partially explained by fish abundance (Fig. 3 column 1). For both regions of the parameter space with vitamin B_1_-poor planktivorous fish (Fig. 2a), a low biomass of mesozooplankton was associated with low fluctuations in populations of picoalgae and mesozooplankton prey (i.e., a low coefficient of variation for the biomass of ciliates and microalgae) (Fig. 3 columns 2–4). Picoalgae biomass drops in our model only if overgrazed by numerous nanoflagellates, which in turn triggers a pulse of ciliates which are in turn exploited by mesozooplankton (Fig. 2b,c). A drop in the biomass of picoalgae, the most competitive class of primary producers, also has also a positive effect on the biomass of microalgae (Fig. 2b,c) as it increases the availability of a significant amount of nutrients and improves light conditions (see below).

**Figure 4:**
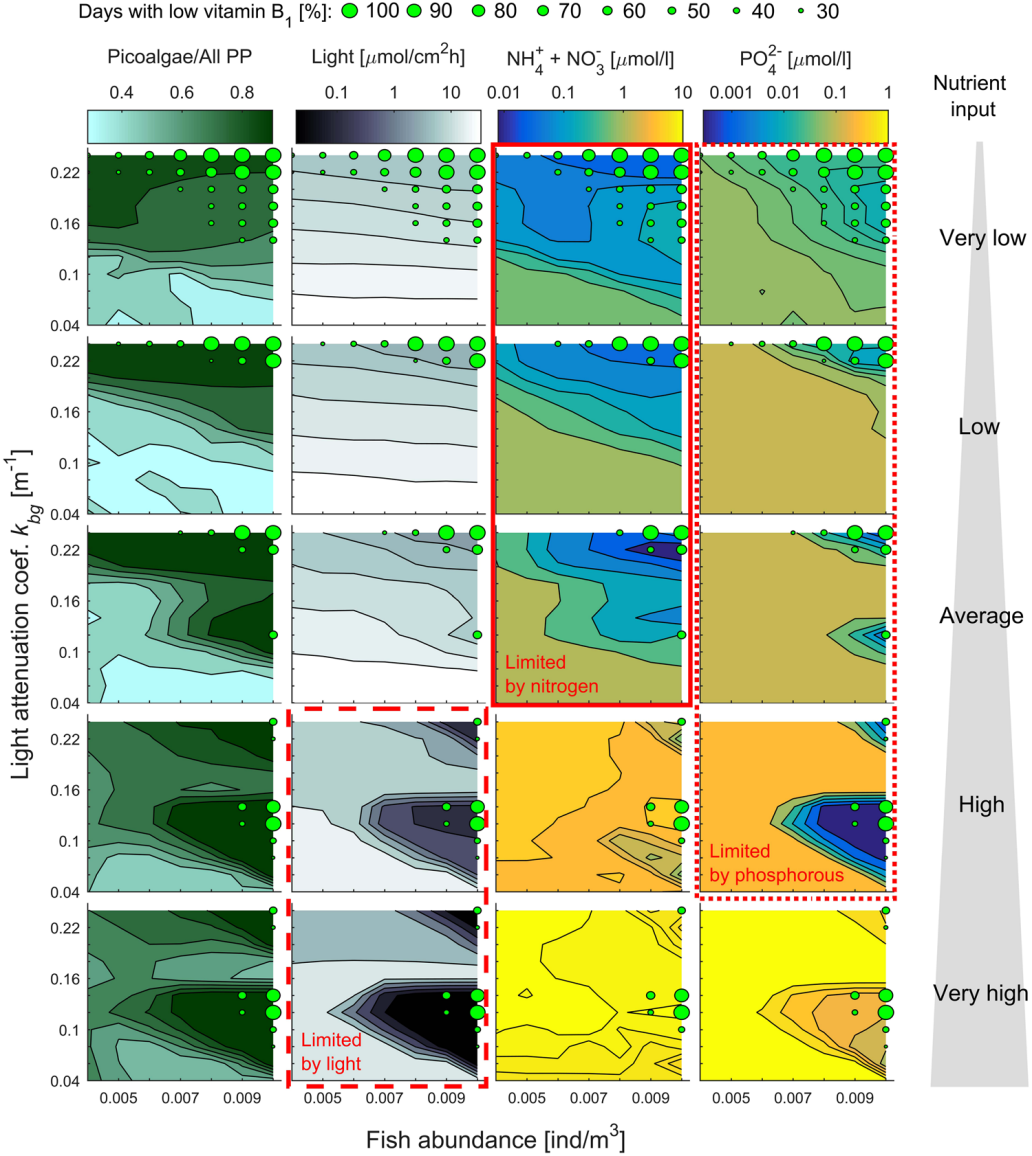
Biotic and abiotic correlates of low vitamin B_1_ levels in planktivorous fish presented in gradients of fish abundance, background light attenuation and nutrient input (illustrated by the gray triangular shape). Cases with vitamin B_1_-poor planktivorous fish are indicated by green circles (see the legend). (Column 1) The average proportion of algae biomass located in picoalgae illustrates the degree to which the primary producer cell-size spectrum shifts toward the smallest primary producers. (Column 2) Total light penetrating the water column (median) from attenuation by primary producers (self-shading) and dissolved substances (background attenuation). Note, that the light attenuation is driven by background attenuation in scenarios with very low to average nutrient input and almost entirely by self-shading in scenarios with high and very high nutrient input. (Columns 3–4) Concentration of dissolved inorganic nitrogen and phosphorous (median). (Columns 2–4) Red rectangles outline the scenarios in which low levels of nitrogen, phosphorous and/or light fit with the observed low levels of vitamin B_1_ in planktivorous fish.

Both scenarios resulting in vitamin B_1_-poor planktivorous fish are associated with a skewed size spectrum of primary producers toward picoalgae (Fig. 4 column 1). However, the underlying mechanisms responsible for the skewed algae size distribution are different in the described scenarios. Under scenario (i), i.e., high fish abundance, high to very high nutrient input and intermediate light attenuation (Fig. 2a), the picoalgae bloom is long-lasting (Fig. 2e). The high biomass of picoalgae drains out phosphorous and causes severe light deprivation (Fig. 4, columns 2 and 4). Hence, under a prolonged picoalgae bloom microalgae growth is suppressed due to the low availability of phosphorus and light (high nutrient input) or of only light (very high nutrient input) (Fig. 4 columns 2 and 4). Under scenario (ii), i.e., intermediate to high fish abundance, very low to average nutrient input, and strong background attenuation (Fig. 2a,d), the growth rate of microalgae is limited by the low availability of nutrients due to the strong competitive abilities of picoalgae that drain out nitrogen and phosphorous (Figs 2d and 4 columns 3–4).

In our model, a large fraction of vitamin B_1_ is degraded during consumption due to its bioavailability *b* = 0.15. Let us consider a hypothetic pool *c* of a compound synthesized by picoalgae. While flowing through the modeled food web (Fig. 1), the pool degrades at every trophic level and as a result, the amount of the compound drops in a negative exponential fashion given by *cb*^*n*^, where *n* denotes the number of consumer levels. The amount of compound *c* will drop 6.6 times after being absorbed by nanoflagellates and ca. 2 thousand times after being absorbed by planktivorous fish, as it needs to pass three consumer levels. Whereas only 0.5‰ of vitamin B_1_ produced by picoalgae can reach planktivorous fish, this value increases to ca. 2% for the pool produced by microalgae, as there are two instead of four consumer levels. Changes in bioavailability significantly affect the predicted amount of vitamin B_1_ that is left after passing subsequent trophic levels. For instance, by reducing bioavailability from 15% to 10%, the vitamin B_1_ pool synthesized by picoalgae will drop 10 thousand times, instead of 2 thousand times, when absorbed by planktivorous fish. Hence, the assumed bioavailability of vitamin B_1_ influenced the number of scenarios with vitamin B_1_-poor planktivorous fish (Fig. S3 in SI Appendix 1). However, even under relatively high vitamin B_1_ bioavailability scenarios, vitamin B_1_-deficient fish persist in two separate regions of the parameter space (see Fig. S3 in SI Appendix 1).

## Discussion

Our work shows, using the Baltic Sea as a model system, that vitamin B_1_ flow in aquatic systems is mediated by processes that control phytoplankton diversity and abundance. Size shifts in primary producers toward picoalgae accompanied by a high abundance of small planktivorous fish lead to low vitamin B_1_ concentrations in the small pelagic fish and therefore to a high risk of vitamin B_1_ deficiencies in top consumers. A limited vitamin B_1_ concentration at the higher levels of the food chain is most likely to occur when there is either a high input of nutrients or a high concentration of dissolved substances that attenuates light. In addition, the sensitivity analysis revealed that one of the crucial aspects for understanding the causes of vitamin B_1_ deficiency in marine ecosystems, to date poorly covered in the literature, is vitamin B_1_ bioavailability, i.e., the proportion of the compound absorbed from food^8,9,34,35,58^.

Administration of vitamin B_1_ to animals that suffer from deficiency syndromes can reverse negative effects; fry of lake trout and Atlantic salmon as well as seabirds suffering from vitamin B_1_ deficiency syndrome recovered when administrated physiological concentrations of vitamin B_1_^8,14,58,59^. Unicellular taxa producing or absorbing dissolved vitamin B_1_ (or it precursors) from water differ with respect to mass-specific vitamin B_1_ concentrations, but in general, they synthesize orders of magnitude more vitamin B_1_ than is needed to sustain physiological functions in marine top predators^31,32,60,61^. However, not all vitamin B_1_ in phytoplankton is transferred to zooplankton since grazing can be constrained. For example, large filamentous cyanobacteria contain comparably high vitamin B_1_ concentrations, but it is not available to zooplankton since the filaments are too large to be consumed^32^. Moreover, the bioavailability of vitamin B_1_ decreases in water environments due to the high solubility of the vitamin. Due to water-solubility, losses of vitamin B_1_ in fish during digestion can be very high, reaching up to 98%^62^. In mammals, the bioavailability of water-soluble vitamin B_1_ hydrochloride reaches up to 5%, with up to ca. 20% efficiency of assimilation of other vitamin B_1_ analogues^63,64^. In insects, the efficiencies of assimilation of B vitamins can range from 7 to ca. 60%^65^. Our work shows how differences in bioavailability on a single trophic level produce serious consequences when the full transfer through the food-web is considered (see results and Fig. S3. in SI Appendix 1). No data exist about the vitamin B_1_ bioavailability in lower trophic levels, such as protozoans or zooplankton, and there is a need for studies that compare the vitamin B_1_ bioavailability at various trophic levels of the marine food webs.

Heterotrophic prokaryotes and photoautotrophs are the main producers of vitamin B_1_ in marine systems^18^. However, a large proportion of bacteria and phytoplankton are auxotrophic, as they rely on an exogenous supply of vitamin B_1_ or its precursors^6,7,18–22,30,66^. The degree of auxotrophy in some groups, such as bacterioplankton, is very high^7^. Vitamin B_1_, due to its high water solubility, cannot be stored in other forms than those built in cellular structures, which makes auxotrophs and consumers to dependent on a continuous intake of vitamin B_1_^67,68^. Concentrations of dissolved vitamin B_1_ and its precursors and degradation products in the water column are not well known, but when measured, they tend to be undetectable or in the picomolar range^18,25^. In our work, we did not consider auxotrophy or complex trophic interactions in order to keep the model general and our understanding of the results feasible. Our work focuses on the transfer of vitamin B_1_ from unicellular taxa up the food chain and the conclusions from our work regarding the transfer to consumers do not change after manipulation of the rates of vitamin B_1_ synthesis/absorption, bioavailability or initial cellular concentrations (see Figs S1–S3 in SI Appendix 1). However, the degree of auxotrophy is likely to affect the rate at which levels of dissolved vitamin B_1_ and microbial species composition fluctuate over time. The dynamics of dissolved and particulate-bound vitamin B_1_ in the microbial realm are currently receiving much attention in the literature, but the overall mechanisms regulating these dynamics are not fully understood^7,18–24,26–30,66,69^. Future models of microbial realm should incorporate these dynamics when the mechanisms regulating microbial cycling of vitamin B_1_ are understood in more detail.

Abiotic stress may cause variation in the vitamin B_1_ content of aquatic primary producers^33^. Our study does not consider the impacts of potential stressors on vitamin B_1_ concentrations, but we do show that large primary producers among phytoplankton are essential for vitamin B_1_ transport through aquatic food webs. Microalgae are in the optimal predator-to-prey size range for mesozooplankton^70^, and they are an important source of vitamin B_1_ for zooplankton^32^. The flow of vitamin B_1_ to the level of top consumers is expected to be more efficient when the compound is coming from microalgae rather than from picoalgae (see results). We cannot exclude the possibility that the constrained flow of vitamin B_1_ to higher trophic levels arises in natural conditions as an effect of abiotic stressors, e.g., pollutants that deplete vitamin B_1_ levels in key groups of producers. However, this hypothesis cannot be tested without a more complete picture of the spatiotemporal variation in the content of vitamin B_1_ in microalgae.

It has been suggested that changes in the diet composition of Atlantic salmon, with a shift from herring to sprat, which contains low levels of vitamin B_1_ per energy unit, is responsible for M74 syndrome in this top predatory fish^13,35^. However, the drivers of spatiotemporal variations in vitamin B_1_ levels in small planktivorous fish are not well understood, and our work indicates that they are likely constituted by both top-down and bottom-up ecosystem mechanisms of regulation. Shifts in abundance and species composition of small planktivorous fish also cannot explain the occurrence of critically low vitamin B_1_ levels reported for bivalves, omnivorous gulls or sea ducks^8,9,14^. Whereas bivalves and common eider ducks represent part of the food web that includes benthic organisms not modeled in our work, we believe that the general predictions of our study may also apply here. A shift toward smaller primary producers and less mesozooplankton, that triggers low vitamin B_1_ levels in planktivorous fish may affect filtrating bivalves in a similar way as the shift impacts planktivorous fish in our model.

Our results, based on the explicit analysis of vitamin B_1_ flow through the food web, point out two aspects that have the potential to balance the current discussion on the drivers of vitamin B_1_ deficiency in aquatic ecosystems. First, the factors affecting the biomass of key groups of primary producers (e.g., microalgae) may be more important for understanding the causes of vitamin B_1_ deficiency than the interspecific differences in mass-specific levels of vitamin B_1_. However, little is known about the degree to which primary producers within a size class may differ with respect to vitamin B_1_ content. Second, there is a great need for studies on taxa-specific differences in vitamin B_1_ bioavailability. Without this knowledge, it is difficult to judge the importance of different parts of the food web to the flow of vitamin B_1_ from producers to consumers. Knowledge of group-specific differences in the bioavailability of vitamin B_1_ would help us to identify key taxa among producers and consumers essential for vitamin B_1_ transport through aquatic food webs. To conclude, a comprehensive approach focused on the flow of vitamin B_1_ through an aquatic food web allows the identification of the environmental and biological factors that lead to the constrained flow of vitamin B_1_. Our work identifies microalgae as organisms of key importance for vitamin B_1_ transport from unicellular taxa to top consumers. High nutrient input, strong light attenuation by dissolved substances and a high population density of planktivorous fish promotes restricted flow of vitamin B_1_. Our work identifies anthropogenic pressures and climate-driven factors that may facilitate the occurrence of vitamin B_1_ deficiency syndrome in top consumers. Apart from the high input of nutrients and dissolved substances that attenuate light, fishing pressure on top predators may also contribute to hampered flow of vitamin B_1_ due to the increased abundance of planktivorous fish. Hence, models that attempt to explain the role of vitamin B_1_ deficiency in marine ecosystems should be an integral part of an Ecosystem-Based Management approach toward the sustainable use of living marine resources^40,71^.

## Supplementary information

Appendix 1
