# Supplementary material for: Modeling vitamin B1 transfer to consumers in the aquatic food web
Source: Sci Rep. 2019 Jul 11;9:10045. doi: 10.1038/s41598-019-46422-2 (PMC6624374; doi:10.1038/s41598-019-46422-2)
Supplement: Supplementary file 1 — Appendix 1 [file 41598_2019_46422_MOESM1_ESM.pdf]

Ejsmond MJ, Blackburn N, Fridolfsson E, Haecky P, Andersson A, Casini M, Belgrano A, Hylander S.
Modelling vitamin B_1 transfer to consumers in the aquatic food web.

Appendix 1. Parameterization, description of methods and a sensitivity analysis of the results model to key parameters

Table of contents

1. Factors affecting mass-specific vitamin B_1 levels

2. Vitamin B_1 bioavailability - sensitivity analysis of the model results

3. Nutrient uptake by primary producers

4. Light attenuation and the effect of light on primary production

5. Tested scenarios

a. Nutrient input

b. Planktivorous fish abundance

c. Degree of light attenuation

1. Factors affecting mass-specific vitamin B_1 levels

We assume, that due to turnover rate of mitochondria and other cell structures, vitamin B_1 is degraded with a rate equal to the metabolic rate i.e. M_R=0.001 [h^{-1}] where M_R is the fraction of vitamin B_1 in the cell degraded per hour. Bacteria and algae are assumed to synthesize and /or absorb dissolved vitamin B_1 with a rate twice of the assumed metabolic rate, i.e. the net rate of vitamin B_1 level increase is equal to the metabolic rate. Note that the predictions from our model do not change when the net rate of vitamin B_1 level in cells of microbes is set to 50% or 200% of metabolic rate M_R (Fig. S1). The simulations starts from picomolar concentrations of vitamin B_1 in tissues of the modelled organisms but the predictions derived from our model also do not change when simulations start from maximal allowed levels of vitamin B_1 (Fig. S2). Hence, the model is robust in terms of changes regarding the assumed rate of vitamin B_1 synthesis/uptake as well as initial cellular concentrations of the vitamin (Fig. S1-2).We used the following empirical estimates to set the maximal mass-specific concentration of vitamin B_1 [$\mu mol \cdot \mu mol^{-1}$ C]: bacteria 1.48e-7, picoalgae 1.48e-7, nanoalgae 1.18e-7 and microalgae 1.18e-7, 1.28e-7 for mesozooplakton [1,2]. Small planktivorous fish were allowed to contain at maximum 1.04e-10 [$\mu mol \cdot \mu mol^{-1}$ C] with average concentration of 6.41^{-11} [$\mu mol \cdot \mu mol\ C^{-1}$] according to the vitamin B_1 content in clupeids of the Baltic Sea averaged data for sprat and herring reported by [3]. To recalculate the concentrations of vitamin B_1 in planktivorous fish we assumed carbon mass to constitute 12.5% of fresh body mass [4]. Due to a lack of empirical data on vitamin B_1 levels in protozoans, we set the maximal levels to 1.32e-7 [$\mu mol \cdot \mu mol^{-1}$ C] for nanoflagellates and 1.27e-7 [$\mu mol \cdot \mu mol^{-1}$ C] for ciliates by fitting a linear regression to the log-transformed data on mass specific vitamin B_1 content in other planktonic organisms. The assimilation rate in vitamin B_1 consumers was dependent on prey and predator biomass [$\mu mol\ C \cdot l^{-1}$], the assumed vitamin B_1 bioavailability (see below and in the main text) and the predators volume-specific clearance rates. The clearance rates were set to $1 \cdot 10^{-5}$ [h^{-1}] for protozoans and mesozooplankton while three times lower specific clearance rate was assumed for clupeid fish [5,6].

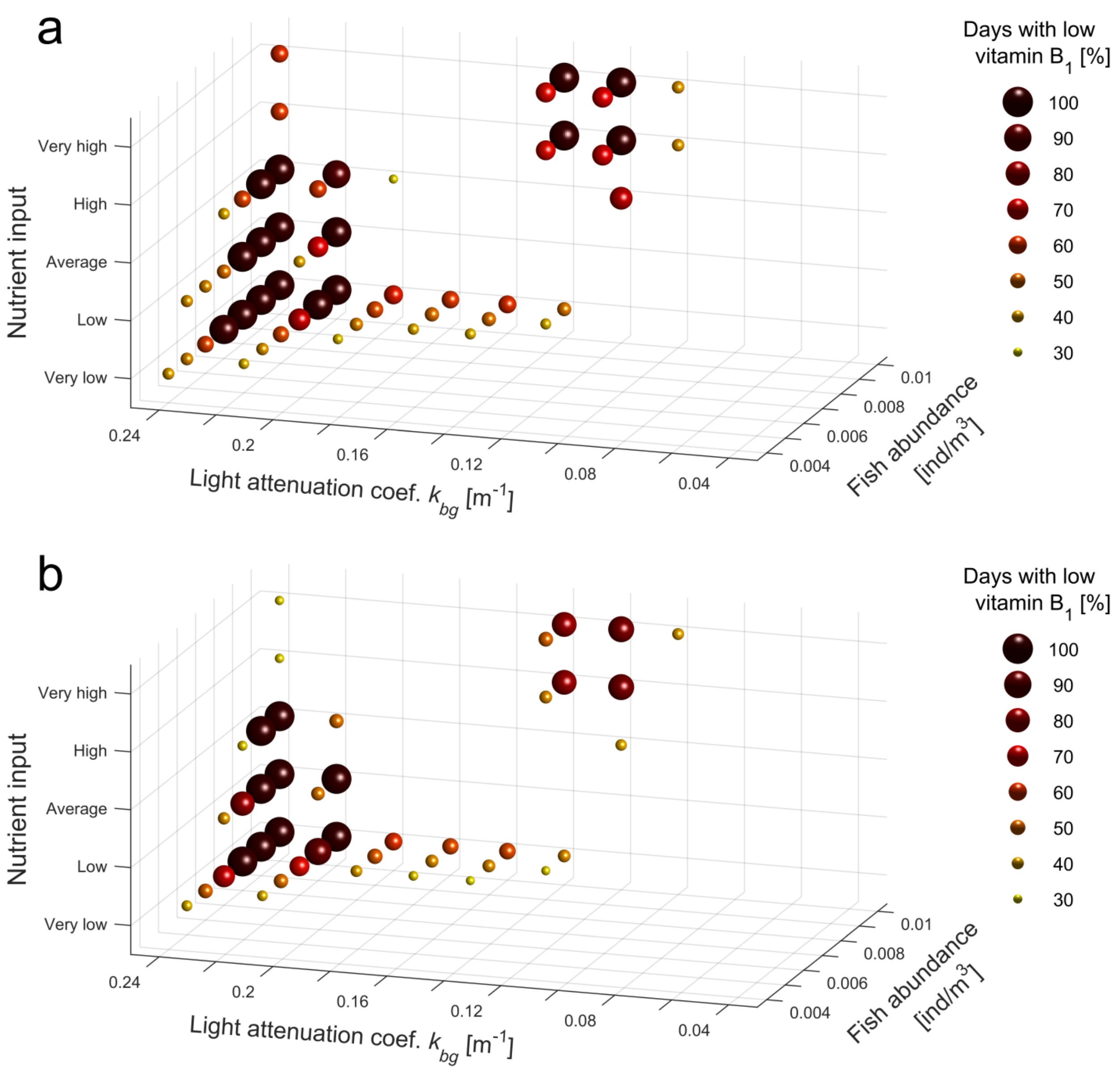

Figure S1. The results of sensitivity analyses of the model predictions where the assumed rate of vitamin B_1 input was set to (a) 50% and (b) 200% of the metabolic rate M_R. Note that all other parameters were set the same as in simulations presented in Fig. 2 in the main text. Spheres represent the percentage of days within the modelled time frame with vitamin B_1 in planktivorous fish falling below the average levels found in empirical studies (see the main text). For clarity spheres indicate scenarios with vitamin B_1 level in planktivorous fish lower than the average empirical estimate in 30% or more days.

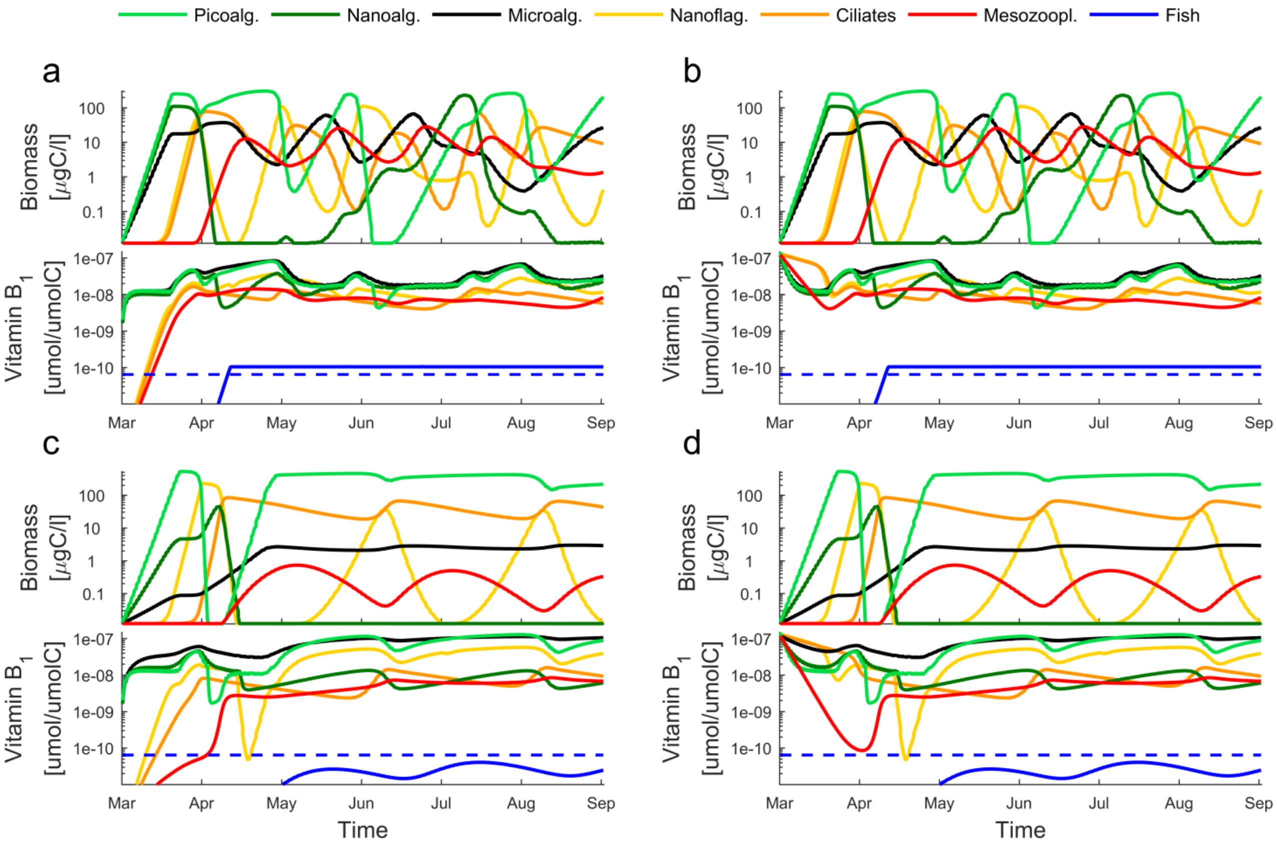

Figure S2. Biomass and concentrations of vitamin B_1 in exemplary scenarios. (a, c) Simulations start from picomolar concentrations of mass specific content of vitamin B_1. (b, d) Simulations start from maximal allowed concentration of vitamin B_1. Other parameters were set the same for pairs of panels a, b and c, d.

2. Vitamin B_1 bioavailability - sensitivity analysis of the model results

The bioavailability of vitamin B_1 determine the fraction of the compound loss during digestion process by consumers. Losses of water-soluble vitamin B_1 in fish during digestion can be very high and reach up to 98% [7]. In mammals bioavailability reaches up to 5% for water-soluble vitamin B_1 hydrochloride and up to ca. 20% for other vitamin B_1 analogues [8,9]. However, no data exist on vitamin B_1 bioavailability in protozoans or zooplankton. In the main text we report results for vitamin B_1 bioavailability of 15% i.e. 75% of the consumed vitamin B_1 is lost. In order to assess the effect of the assumed bioavailability levels on the model outcomes we run a sensitivity analysis. We ran calculations in the full space of model parameters i.e. abundance of planktivorous fish, nutrient input

and light attenuation coefficient caused by dissolved substances with vitamin B_1 bioavailability ranging from 10 to 20% (Fig. S3).

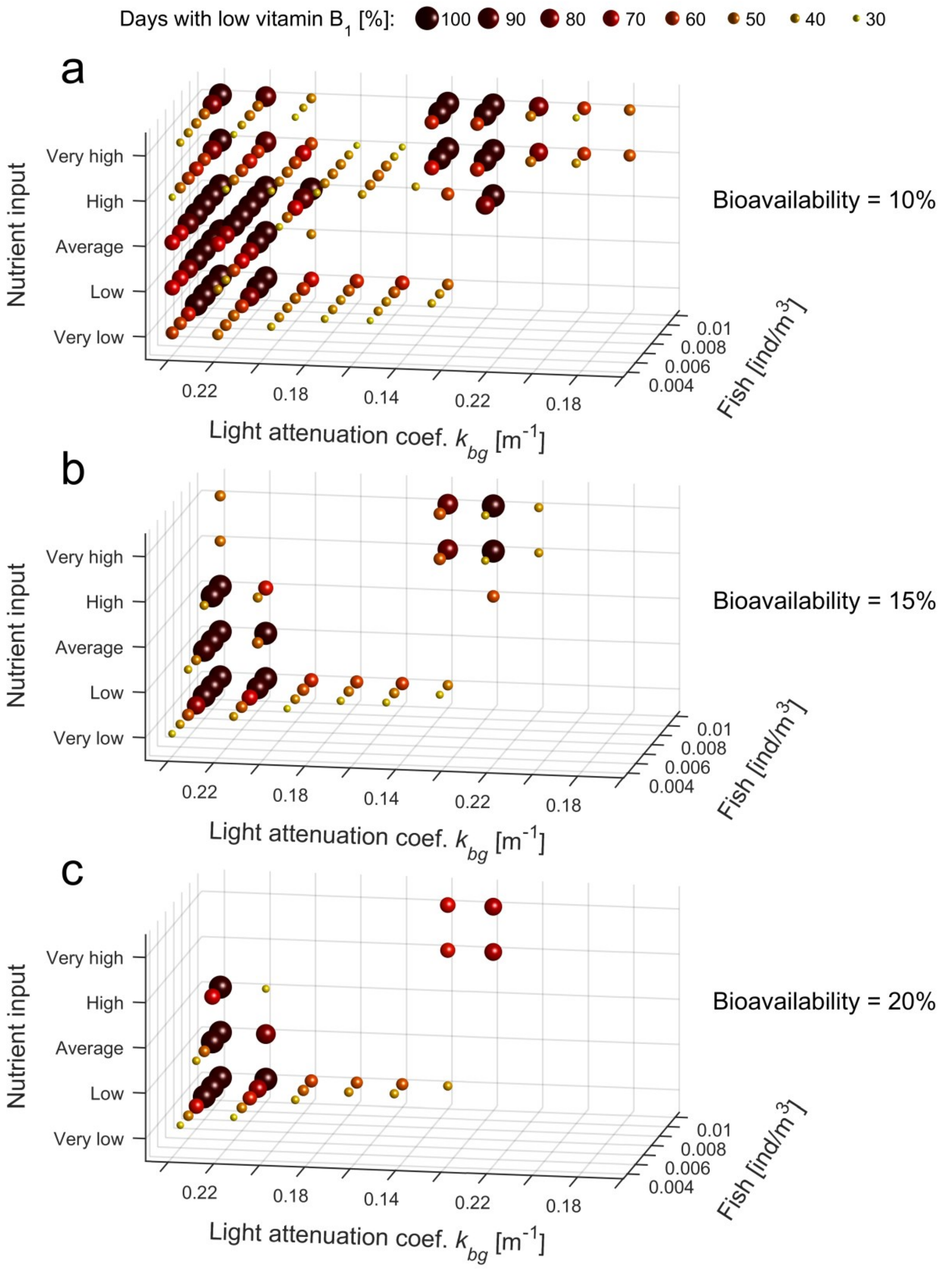

Figure S3. Sensitivity of the model predictions to the assumed bioavailability of vitamin B_1. Spheres represent the percentage of days within the modelled time frame with vitamin B_1 in planktivorous fish falling below the average levels found in empirical studies (see the main text). For clarity spheres

indicate scenarios with vitamin B_1 level in planktivorous fish lower than the average empirical estimate in 30% or more days.

3. Nutrient uptake by primary producers

The rate of nutrient uptake is a meaningful measure of competitive strength in marine and freshwater primary produces [10,11]. In previous studies of aquatic productivity, nutrient transport was modelled as constrained by the rate of the diffusion only cf. [5]. These studies assumed that algae cell is a perfect sink for nutrients and large cells are constrained by nutrient transport rate to a higher degree than small unicellular algae. In our model, consistent in approach with recent literature, nutrient affinity and nutrients-dependent growth rate scales with cell volume rather than its radius [10,11]. We modelled the rate of nutrient transport as allometrically dependent on cell volume using formulas for dependence of nutrient affinity [l·day^{-1}] on cell volume [μm^3] reported by Edwards, et al. [10] and theoretically investigated by Lindemann, et al. [12], given by $J_N = 10^{-5.1} r^{2.25} 24^{-1} C_N$ and $J_P = 10^{-5.5} r^{2.55} 24^{-1} C_P \left[\mu\text{mol} \cdot \text{h}^{-1} \right]$ for nitrogen and phosphorous.

4. Light attenuation and the effect of light on primary production

Light intensity in the model fluctuates in the annual and day-night cycle determined by day of year *d*, hour *h* and latitude *φ* at the geographic location of Linnaeus Microbial Observatory (LMO) at 56.93°N and 17.06°E. The intensity of unidirectional light reaching water surface I_0 [μmol quanta cm^{-2}·h^{-1}] is given by

(1) $I_0 = I_S \sin\alpha$

where I_S match the daytime photosynthetically active radiation, set to I_S=100 [μmol quanta m^{-2}·s^{-1}] cf. [5,13] and *α* match the elevation angle. The elevation angle is given by

(2) $\alpha = \arcsin\left(\sin\delta \sin\varphi + \cos\delta \cos\varphi \cos\theta\right)$

with hour angle given by and $\theta = 15(h-12)$ and declination *δ* equal to

$$(3)\ \delta = 23.45^{\circ} \sin\left(\frac{360}{365}(d-81)\right)$$

Consequently, I_0 was set to 0 in between after sunset and before sunrise hours.

The light availability is one of key determinants of the growth rate of primary producers (see below). The primary production in our model *PP* [μmol C·h^{-1}] was dependent on absorption of light that attenuates while penetrating the 10m water layer. The way we model light attenuation follows the approach based on the Lambert-Beer's law, adopted in several studies on algae primary production e.g. [14,15,16], and given by

$$(4)\ I(W_{PP}, z) = I_0 \exp(-kz)$$

where I_0 is the intensity of light reaching water surface, *z* is depth in meters and *k* match the light attenuation coefficient [m^{-1}]. The light attenuates due to self-shading by algae and background sources. The total attenuation coefficient *k* given by

$$(5)\ k = k_s W_{PP} + k_{bg}$$

is determined by background attenuation coefficient k_{bg} [m^{-1}], algal carbon biomass W_{PP} [mg C·m^{-3}], and carbon mass specific attenuation coefficient k_s. We set a carbon mass specific attenuation coefficient k_s to $3\cdot10^{-4}$ [m^2·mg C^{-1}] which is consistent with values set in other studies on marine primary production cf. [14,16] and estimates for the Baltic Sea based on chlorophyll concentration cf. [17,18]. We use background attenuation coefficient k_{bg} to model scenarios with variable degree of light attenuation by organic and inorganic substances dissolved in the water (see below).

The carbon production by an algae cell in our model *PP* [μmol C·h^{-1}] depends on total light penetrating a layer of surface waters with depth *z*=10 [m] and is given by

$$(6)\ PP = \Phi \pi r^2 I$$

where πr^2 is the light-exposed surface of the cell area [cm^2], total light penetrating the water column *I* [μmol quanta cm^{-2}·h^{-1}] and Φ is the quantum yield of photosynthesis [mol C·mol quanta^{-1}] parameterized to Φ=0.0286 [mol C·mol quanta^{-1}] according to estimates of Φ_{max} for the spring and summer bloom in the Baltic Sea data taken from [19,20].

5. Tested scenarios

a.) Nutrient input

The degree to which nutrient concentrations and nutrient ratios constrain the growth rate of primary producers depends not only on the nutrient concentration in the water but also on the optimal stoichiometric composition of cells. Marine primary producers are highly variable with respect to their stoichiometric composition, with C:N:P ratio distributed around the Redfield ratio i.e. 106:16:1 [21]. Because our understanding of the adaptive component of this variability is poor [22,23] and to keep our model simple we assumed that primary producers are equally constrained by nitrogen and phosphorous availability. Hence, we assumed in our model that N:P ratio of algae cells and dissolved nutrients follows the Redfield ratio cf. [5] but we manipulated the level of nutrients available in the water at the beginning of simulation. We parameterized concentration of nitrate NO_3^- and ammonium NH_4^+ using data obtained at the Linnaeus Microbial Observatory (LMO) sampling site [24] and data extracted from HELCOM database representing early spring nutrient concentration in the southern part of the Baltic Sea (years 1998-2016). We consider a set of five scenarios varying with nitrogen input from a very low to a very high nitrogen concentration (2.01 µmol/l of NO_3^-, 0.93 µmol/l of NH_4^+ and 30 µmol/l of NO_3^-, 7 µmol/l of NH_4^+) with levels of phosphorous input set relatively to nitrogen levels according to the Redfield ratio i.e. 16:1. Consequently, in the model we consider the following scenarios of the starting concentration of nitrogen and phosphorous: very low (2.01 µmol/l of NO_3^-, 0.93 µmol/l of NH_4^+, 0.18 PO_4^{2-}), low (2.92 µmol/l of NO_3^-, 2.05 µmol/l of NH_4^+, 0.31 PO_4^{2-}), average (3.82 µmol/l of NO_3^-, 3.17 µmol/l of NH_4^+, 0.44 PO_4^{2-}), high (16.91 µmol/l of NO_3^-, 5.09 µmol/l of NH_4^+, 1.37 PO_4^{2-}), very high (30.00 µmol/l of NO_3^-, 7.00 µmol/l of NH_4^+, 2.31 PO_4^{2-}). We also rounded the precision of the nutrient concentration to two decimal places so it can be easily compared with levels measured in natural environments.

b.) Planktivorous fish abundance

We modelled population of planktivorous fish with body mass 4 g carbon body weight equivalent to ca. 32 g of fresh weight and ca. 15cm body length cf. [4], which is an intermediate body size between

those for adult Baltic herring (*Clupea harengus membras*) and European sprat (*Sprattus sprattus*) see. [25,26]. We used the data on abundance of central Baltic population of herring and sprat in age 1+ in years 1991-2016 ICES subdivisions 25-28 see [27] to parameterize tested scenarios with respect to the abundance of planktivorous fish. The averaged population densities of herring and sprat in the Baltic Sea subdivisions 25-28 varied between 0.004 and c.a. 0.05 [ind·m^{-3}] [27,]Data from the Baltic International Acoustic Survey, BIAS: [28]. The density distribution of sprat and herring cumulative abundance of from subdivisions 25-28 in years 1991-2016 was characterized by quartiles $Q_{25\%}$=0.008 and $Q_{75\%}$=0.02 [ind·km^{-3}] (Fig. S4). Our model assumes fish predate on mesozooplankton continuously and we did not model diel vertical migrations in zooplankton and associated with this day-night cycle in fish feeding intensity. Hence, the abundance of planktivorous fish in the model was proportional to the empirical estimate of clupeids abundance (Fig. S4) by dividing real abundances by half. This transformation of empirically estimated abundances into modelled abundances represent the fact that in natural environments predation intensity fluctuates in day-night cycle, which is not the case in our model. Consequently, we tested our model with scenarios varying in planktivorous fish population density from low (0.004 [ind·m^{-3}]) to high (0.01 [ind·m^{-3}]) abundance (see the main text).

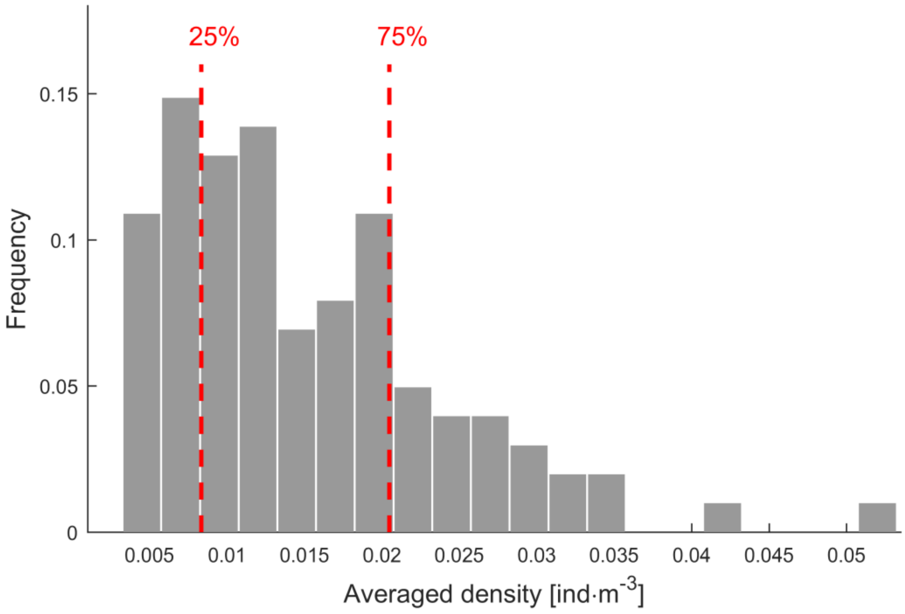

Figure S4. Frequency distribution of the two key clupeid species abundance in the Baltic Sea. Bars represent averaged population density for Baltic herring (*Clupea harengus membras*) and European sprat (*Sprattus sprattus*) in age 1+ in the Baltic Sea (subdivisions 25-28) between 1991 and 2016y. Red dashed lines represent quartiles $Q_{25\%}$ and $Q_{75\%}$.

c. Degree of light attenuation

The light attenuation in our model has a biotic component due to self-shading and an abiotic component set by the background light attenuation k_{bg} see eq. (5). Whereas light attenuation due to primary production is determined by the biomass of algae see eq. (5) the background attenuation coefficient k_{bg} describes light attenuation by dissolved inorganic and organic substances. The background attenuation coefficient k_{bg} for marine systems reported by Urtizberea, et al. [29] served as an empirical estimate for the scenarios tested in our work. We tested a range of scenarios varying in k_{bg} coefficient from 0.04 [m^{-1}] to 0.24 [m^{-1}].

6. References

1 Fridolfsson, E. *et al.* Seasonal variation and species-specific concentrations of the essential vitamin B_1 (thiamin) in zooplankton and seston. *Marine Biology* (in press).

2 Fridolfsson, E., Lindehoff, E., Legrand, C. & Hylander, S. Thiamin (vitamin B1) content in phytoplankton and zooplankton in the presence of filamentous cyanobacteria. *Limnology and Oceanography* **63**, 2423-2435, doi:doi:10.1002/lno.10949 (2018).

3 Keinanen, M. *et al.* The thiamine deficiency syndrome M74, a reproductive disorder of Atlantic salmon (Salmo salar) feeding in the Baltic Sea, is related to the fat and thiamine content of prey fish. *Ices Journal of Marine Science* **69**, 516-528, doi:10.1093/icesjms/fss041 (2012).

4 Arrhenius, F. & Hansson, S. Growth and seasonal changes in energy content of young Baltic Sea herring (Clupea harengus L). *Ices Journal of Marine Science* **53**, 792-801, doi:10.1006/jmsc.1996.0100 (1996).

5 Thelaus, J., Haecky, P., Forsman, M. & Andersson, A. Predation pressure on bacteria increases along aquatic productivity gradients. *Aquatic Microbial Ecology* **52**, 45-55, doi:10.3354/ame01200 (2008).

6 Kiorboe, T. & Hirst, A. G. Shifts in Mass Scaling of Respiration, Feeding, and Growth Rates across Life-Form Transitions in Marine Pelagic Organisms. *American Naturalist* **183**, E118-E130, doi:10.1086/675241 (2014).

7 Tacon, A. G. J. *Nutritional fish pathology. Morphological signs of nutrient deficiency and toxicity in farmed fish.*, Vol. 330 (FAO, 1992).

8 Loew, D. Pharmacokinetics of thiamine derivatives especially of benfotiamine. *International Journal of Clinical Pharmacology and Therapeutics* **34**, 47-50 (1996).

9 Tallaksen, C. M. E., Sande, A., Bohmer, T., Bell, H. & Karlsen, J. Kinetics of thiamin and thiamin phosphate esters in human blood, plasma and urine after 50 mg intravenously or orally. *European Journal of Clinical Pharmacology* **44**, 73-78, doi:10.1007/bf00315284 (1993).

10 Edwards, K. F., Thomas, M. K., Klausmeier, C. A. & Litchman, E. Allometric scaling and taxonomic variation in nutrient utilization traits and maximum growth rate of phytoplankton. *Limnology and Oceanography* **57**, 554-566, doi:10.4319/lo.2012.57.2.0554 (2012).

11 Fiksen, O., Follows, M. J. & Aksnes, D. L. Trait-based models of nutrient uptake in microbes extend the Michaelis-Menten framework. *Limnology and Oceanography* **58**, 193-202, doi:10.4319/lo.2013.58.1.0193 (2013).

12 Lindemann, C., Fiksen, Ø., Andersen, K. H. & Aksnes, D. L. Scaling Laws in Phytoplankton Nutrient Uptake Affinity. *Frontiers in Marine Science* **3**, doi:10.3389/fmars.2016.00026 (2016).

13 Bengtsson, B. *et al.* Reproductive Disturbances in Baltic Fish: A Synopsis of the FiRe Project. *AMBIO* **28**, 2-8 (1999).

14 Hall, S. R., Leibold, M. A., Lytle, D. A. & Smith, V. H. Grazers, producer stoichiometry, and the light : nutrient hypothesis revisited. *Ecology* **88**, 1142-1152, doi:10.1890/06-0923 (2007).

15 Huisman, J. & Weissing, F. J. Competition for Nutrients and Light in a Mixed Water Column: A Theoretical Analysis. *American Naturalist* **146**, 536-564, doi:10.1086/285814 (1995).

16 Jager, C. G., Diehl, S. & Emans, M. Physical Determinants of Phytoplankton Production, Algal Stoichiometry, and Vertical Nutrient Fluxes. *American Naturalist* **175**, E91-E104, doi:10.1086/650728 (2010).

17 Ficek, D. *et al.* Spectra of light absorption by phytoplankton pigments in the Baltic; conclusions to be drawn from a Gaussian analysis of empirical data. *Oceanologia* **46**, 533-555 (2004).

18 Meler, J., Ostrowska, M., Ficek, D. & Zdun, A. Light absorption by phytoplankton in the southern Baltic and Pomeranian lakes: mathematical expressions for remote sensing applications. *Oceanologia* **59**, 195-212, doi:10.1016/j.oceano.2017.03.010 (2017).

19 Raateoja, M., Seppala, J. & Kuosa, H. Bio-optical modelling of primary production in the SW Finnish coastal zone, Baltic Sea: fast repetition rate fluorometry in Case 2 waters. *Marine Ecology Progress Series* **267**, 9-26, doi:10.3354/meps267009 (2004).

20 Ficek, D. *et al.* Practical applications of the multi-component marine photosynthesis model (MCM). *Oceanologia* **45**, 395-423 (2003).

21 Geider, R. J. & La Roche, J. Redfield revisited: variability of C : N : P in marine microalgae and its biochemical basis. *European Journal of Phycology* **37**, 1-17, doi:10.1017/s0967026201003456 (2002).

22 Klausmeier, C. A., Litchman, E., Daufresne, T. & Levin, S. A. Optimal nitrogen-to-phosphorus stoichiometry of phytoplankton. *Nature* **429**, 171-174, doi:10.1038/nature02454 (2004).

23 Sardans, J., Rivas-Ubach, A. & Penuelas, J. The elemental stoichiometry of aquatic and terrestrial ecosystems and its relationships with organismic lifestyle and ecosystem structure and function: a review and perspectives. *Biogeochemistry* **111**, 1-39, doi:10.1007/s10533-011-9640-9 (2012).

24 Legrand, C. *et al.* Interannual variability of phyto-bacterioplankton biomass and production in coastal and offshore waters of the Baltic Sea. *AMBIO* **44**, 427-438, doi:10.1007/s13280-015-0662-8 (2015).

25 Casini, M., Cardinale, M. & Arrhenius, F. Feeding preferences of herring (Clupea harengus) and sprat (Sprattus sprattus) in the southern Baltic Sea. *Ices Journal of Marine Science* **61**, 1267-1277, doi:10.1016/j.icesjms.2003.12.011 (2004).

26 ICES. *http://www.ices.dk/marine-data/maps/Pages/ICES-FishMap.aspx* (FishMap).

27 Casini, M. *et al.* Spatial and temporal density dependence regulates the condition of central Baltic Sea clupeids: compelling evidence using an extensive international acoustic survey. *Population Ecology* **53**, 511-523, doi:10.1007/s10144-011-0269-2 (2011).

28 ICES. *Final Report of the Baltic International Fish Survey Working Group. WGBIFS Report*. Vol. 27-31 March 2017 (Riga, Latvia., 2017).

29 Urtizberea, A., Dupont, N., Rosland, R. & Aksnes, D. L. Sensitivity of euphotic zone properties to CDOM variations in marine ecosystem models. *Ecological Modelling* **256**, 16-22, doi:10.1016/j.ecolmodel.2013.02.010 (2013).
